# Secure and Blockchain-Based Emergency Driven Message Protocol for 5G Enabled Vehicular Edge Computing

**DOI:** 10.3390/s20010154

**Published:** 2019-12-25

**Authors:** Lewis Nkenyereye, Bayu Adhi Tama, Muhammad K. Shahzad, Yoon-Ho Choi

**Affiliations:** 1 Department of Computer and Information Security, Sejong University, Seoul 05006, Korea; nkenyele@sejong.ac.kr; 2 Department of Mechanical Engineering, Pohang University of Science and Technology, Pohang 37673, Korea; btama@acm.org; 3 Department of Computing, National University of Sciences and Technology (NUST), Islamabad 44000, Pakistan; mkhuram.shahzad@seecs.edu.pk; 4 School of Computer Science and Engineering, Pusan National University, Busan 46241, Korea

**Keywords:** vehicle edge computing, 5G cellular networks, blockchain, multi-receiver signcryption, security, privacy

## Abstract

Basic safety message (BSM) are messages that contain core elements of a vehicle such as vehicle’s size, position, speed, acceleration and others. BSM are lightweight messages that can be regularly broadcast by the vehicles to enable a variety of applications. On the other hand, event-driven message (EDM) are messages generated at the time of occurrence such as accidents or roads sliding and can contain much more heavy elements including pictures, audio or videos. Security, architecture and communication solutions for BSM use cases have been largely documented on in the literature contrary to EDM due to several concerns such as the variant size of EDM, the appropriate architecture along with latency, privacy and security. In this paper, we propose a secure and blockchain based EDM protocol for 5G enabled vehicular edge computing. To offer scalability and latency for the proposed scenario, we adopt a 5G cellular architecture due to its projected features compared to 4G tong-term evaluation (LTE) for vehicular communications. We consider edge computing to provide local processing of EDM that can improve the response time of public agencies (ambulances or rescue teams) that may intervene to the scene. We make use of lightweight multi-receiver signcryption scheme without pairing that offers low time consuming operations, security, privacy and access control. EDM records need to be kept into a distributed system which can guarantee reliability and auditability of EDM. To achieve this, we construct a private blockchain based on the edge nodes to store EDM records. The performance analysis of the proposed protocol confirms its efficiency.

## 1. Introduction

Abrupt situations on roads, such as car accidents, slippery roads, or land sliding can be reported by the vehicles using the inbuilt sensors and devices. Reporting emergency situations can be an effective approach in situations where the evident proofs are required such as car accidents or reckless driving. Those reports can also provide additional materials like pictures, videos, audios to the rescue departments for an efficient and timely intervention [1]. Emergency warning can be divided in three categories; (1) a vehicle-to-vehicle (V2V) warning dissemination such as hazardous or slippery road where the vehicles in the vicinity need to slow down or take further precautions, (2) vehicle-to-infrastructure (V2I) warning such as car accidents or road sliding where the rescue teams (police or medical teams) can use the multimedia proof for an effective and timely response, (3) the last warning dissemination is the combination of the two scenarios. We do focus on the second scenario in this work and those files are called event-driven messages (EDM). Moreover, the EDM files would be sent to remote servers which will require a heavy bandwidth with excessive response delay. In big cities with millions of vehicles running on the road every day, the amount of data to be processed would be massive [2,3,4].

Edge computing was introduced as a new paradigm that takes the computing tasks to the network edge. The edge nodes collect data from the vehicles and process them rather than sending them to a central cloud server. This paradigm offers a number of benefits such as geo-distribution and low latency and can be applied in vehicular networks to offer real time services such as emergency warning, road surface monitoring and navigations [5,6,7]. However, despite the merits of edge computing in vehicular communications, the proposed solutions and applications have not been deployed worldwide due to lack of scalability and adequate communication supports. Recently, the researchers have raised the limitations of IEEE 802.11p due to its lacks of mobility support, also the long-term evolution (LTE) of fourth generation networks (4G) cannot offer effective latency that suits the vehicular networks based applications [8,9,10]. To overcome these limitations, 5G cellular networks were adopted as the ultimate architecture which could help a real deployment of vehicular based technologies. For instance, Uber made a successful test of driverless vehicle using 5G cellular networks recently. The cellular networks offer higher mobility support, reduced latency and massive connectivity that are core requirements for vehicular applications [11,12,13]. Meanwhile, security and privacy issues are also a considerable concern for vehicular communications. For example, vehicles reporting the emergency warnings might not need to disclose their locations expect to authorized third parties. The identities of the vehicles participating in the sending the warnings, their destinations and itinerary are highly sensitive information that need to be carefully handled. Currently, the misuse of vehicles’ users data have been reported massively in the press where untrusted third parties and malicious users leaked the vehicles’ sensitive data [14].

In the literature, a considerable number of articles has been published on secure message dissemination for vehicle networks and can be categorized in three groups: (1) Security for beacons messages also knows as basic safety message (BSM) for the US. These are periodic messages containing vehicle’s position, speed and direction, etc. [15,16,17]; (2) event-driven message (EDM) that are generated at the time of occurrence such as accident alerts or emergency reports [18]. There are many solutions that addressed BSM based scenarios for privacy and security as shown in this recent survey [19]. However, these schemes cannot be directly applied to EDM scenarios for the following reasons. First, the schemes are not built based on 5G-enabled architecture which offers low latency and mobility support for vehicular communications. Second, the current literature such as [20] mainly suggests anonymous authentication schemes and message encryption for secure communications and the central cloud or edge nodes are mainly supposed to be secure. However, if the central cloud is compromised, the rescue services can not retrieve the important files needed for their services, thus data auditability and reliability is very crucial and one of the solutions to achieve data auditability would be through a private blockchain maintained by the edge devices [21,22]. Third, most of the schemes is built using expensive bilinear pairing techniques which are expensive and time consuming operations that degrade the overall protocol performance. To the best of our knowledge, there is one relevant article for emergency message dissemination for vehicular communications [23]. The authors basically presented a fog assisted architecture, highlighted limitations of relevant schemes in the literature and concluded with open research discussions.

Thus, a privacy preserving, secure yet auditable protocol for emergency message in vehicle edge computing is appealing. The contributions of this paper are three folds:

- We describe a novel architecture for emergency warning dissemination using edge computing and private blockchain. The proposed architectures uses 5G network technologies for communication. In our model, we design a secure and privacy preserving model that protect the sensitive data (identity, location, shared data, etc) of the vehicles participating in emergency warning dissemination. We assume that the edge nodes and cloud are semi trusted, therefore our architecture proposes a private blockchain using edge nodes to record the EDM in an immutable and verifiable ledger to guarantee EDMs auditability.
- We design a secure and blockchain based EDM protocol for 5G enabled vehicle edge computing using the private blockchain technique to provide EDM auditability. We make use of lightweight multi-receiver signcryption scheme without pairing that offer low time consuming operations, security, privacy and access control.
- We provide an analysis of security and privacy features of the proposed protocol and evaluation in respect of private blockchain construction, computational and communication costs.

The remainder of this paper is as follows. We review the related work on 5G enabled vehicular edge computing and secure emergency message dissemination in Section 2. We design the system model and the preliminaries of the core cryptographic schemes used in our protocol in Section 3. Section 4 describes the proposed scheme and we provide security, privacy and performance analysis in Section 5. Finally, the concluding remarks are given in Section 6.

## 2. Related Work

This section first describes the basic concepts of vehicular edge computing, then outlines the basic notions of private blockchain technology and concludes with a review of the current schemes on EDM schemes in vehicular networks.

### 2.1. 5G Enabled Vehicular Edge Computing

Vehicular edge computing (VEC) or vehicular edge computing networks (VECONs) are extended from the conventional VANETs. The main difference is that VEC are made by an additional edge layer [24,25]. VEC is basically made by three layers, first the vehicle layer where the embedded sensors in the vehicle collect the data and send them to the edge layer using the onboard unit (OBU). The edge layer is a cluster made by several roadside units (RSUs) within a given distance. The RSU keeps or processes the data provided by the vehicles in the edge cluster. The following layer is called the cloud layer that manages the edge layers. The cloud layer can store massive data and make complex delay tolerant operations on the data provided by the edge layers. The cloud layer can be a data center or intelligent transportation system or regional trusted authority. Although 4G technology can be used, there are inherent drawbacks that has been raised by the research community for an effective vehicular communications networks using 4G technologies. A number of attacks performed on the international subscriber identity for the 4G LTE networks revealed its weakness to provide integrity, non-repudiation, accountability on user data. In addition, IEEE 802.11p can not be relied on for VEC due to its lack of mobility support [8,9,10]. In the forthcoming fifth-generation (5G) cellular-based vehicle networks, the use of denser and smaller cells are anticipated to offer a high transmission rate for the vehicles users. This will enable a range of application starting from the safety related applications to entertainment use cases. The nature of vehicular networks presents a different requirement from the conventional mobile networks. This is basically due to the volatile mobility of the vehicles in the network, the speed of the vehicles along with the topology of the dynamic wireless networks. Therefore, the use of 5G cooperative small cells is discussed in the literature as a promising recommendation [26,27,28]. We adopt in this paper a 5G enabled edge vehicular computing model as underlying network architecture for the proposed protocol.

### 2.2. Blockchain

Blockchain technology is a distributed ledger technique that was first introduced for the financial domain starting with Bitcoin crypto currency. It offers data auditability using authenticated blocks added on the system. It is also a decentralized network since it does not require a centralized entity because it is a peer to peer network [21,22]. In order to add a new block, a consensus algorithm need to be agreed upon by the entities in the chain. Private blockchain was introduced for restricted environment such as businesses or companies where public readability can not be applied. A private blockchain is a network where the participants require a permission to join. In this work, we consider a proof of stake consensus algorithm that randomly choose one of the entities proportionally to each node’s stake to run the process [29].

### 2.3. Secure Schemes for Emergency Warning in VEC

A considerable number of researchers have documented security and privacy related solutions for BSM based scenario as shown in this survey [18]. Nevertheless, these schemes are not directly applicable to EDM scenarios for the following reasons. The protocols do not consider a 5G-enabled architecture which offers low latency and mobility support for vehicular communications. Then, the schemes in the literature such as [20] mainly suggest anonymous authentication techniques and EDM/BSM encryption for secure communications while the central cloud or edge nodes are most of the time supposed to be secure. As mentioned earlier, this raises a huge issue with billions of connected devices, the regional or center cloud or edge devices can be compromised, thus data auditability and reliability need to be taken into consideration. One alternative way of achieving data auditability would be through a private blockchain maintained by the edge devices [21,22]. Also, most of the scheme such as [20] are built using expensive bilinear pairing techniques which are very heavy for mobile and ad hoc networks. In [23], the authors proposed an emergency message dissemination for vehicular communications. Though the paper specifically target EDM, the authors mainly presented fog assisted architecture, highlighted limitations of relevant schemes in the literature and concluded with open research discussions. Their protocol do not address security issues, latency sensitive architecture, distributed environment and EDM reliability and auditability. Thus, we are appealed in this paper to investigate on secure communication for EDM scenario taking into consideration that EDM could be very heavy (heavy videos, audio), while the privacy of the vehicles’ users is not neglected, yet EDM reliability and auditability are guaranteed.

## 3. System Model

In this section we first present the system architecture of the proposed protocol and outline the basic concepts of cryptographic techniques used to construct our protocol.

### 3.1. Main Entities

**Figure 1:**
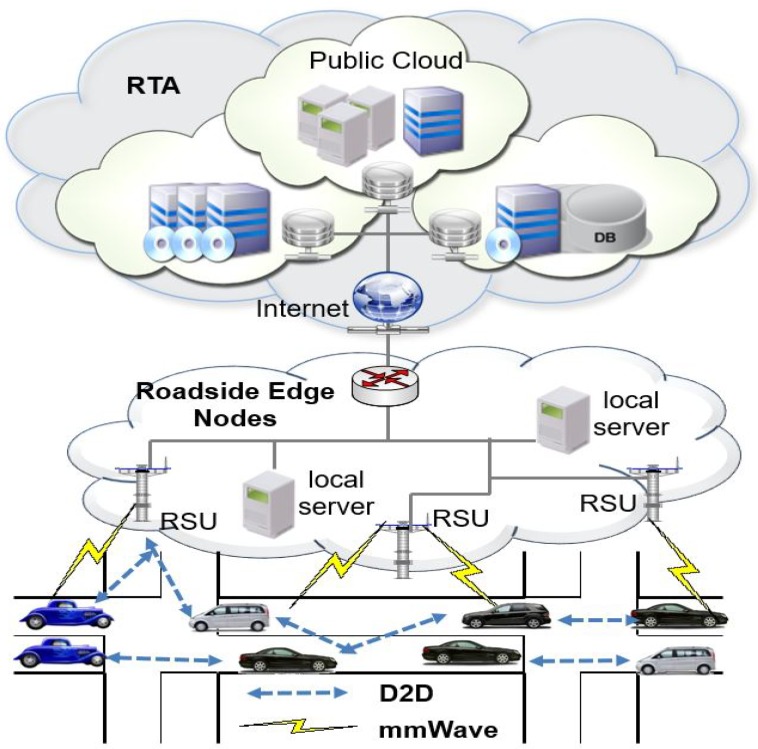
System architecture.

Our proposed system model is made by a main regional overviewer called RTA, the road side units (RSCs) that make a edge cluster and the vehicles that provide EDM files collected through their sensors as shown in Figure 1. We outline the role of each entity in the following:

- Regional Transportation Authority (RTA): RTA is considered as a trusted agency that offers the registration of all the entities within the proposed system (vehicles and edge nodes) and generate cryptographic materials to the entities during the system setup.
- RSU edge nodes: Similar to a sever with limited capabilities, edge nodes are devices placed on the roads with efficient computing, communication and also storage aptitude. Their principal role is the collection of EDM provided by the vehicles, verifies the validity of EDM through designcryption and share the EDM to RTA or any entity that might need the EDM. In real life applications, the EDM could be needed by rescue services such as police or medical centers. We did not explicitly add these entities but we assumed that they have servers in the cloud which are connected to RTA servers as shown in Figure 1. We assume that edge nodes are connected to a source that generate electricity power.
- Vehicles: The vehicles are assumed to be equipped with several sensors and devices such as camera. The onboard units (OBU) in the vehicle gather all the those data in form of EDM files, sends them to edge nodes using different communication means such as D2D or mmWave communications. All vehicles need to register with the RTA at the time of periodic inspection. Besides the well known identifiers of vehicles such as the Electronic License Plate (ELP) or the electronic chassis number (ECN), every vehicle is given a 5G unique identifier (5GID), which is similar to subscriber identification module (SIM) as it is for 3G and 4G cellular networks.

### 3.2. Communication Model

Motivated by the 5G cellular networks architecture, the proposed 5G enabled vehicle edge computing is made by the following components:

- Heterogeneous networks: This network aims at achieving high data rate and network capacity for the 5G-enabled vehicle edge computing. Therefore, two alternative techniques may help to get the mentioned capacities through smaller cells which increase the spectral efficiency. In addition, using the mmWave spectrum would offer high data rates since it operates within the range of 30–300 GHz and 1–10 mm for the spectrum and wavelength respectively [11].
- D2D Communications: D2D communication would enable the vehicles to communicate with each edge device within the licensed cellular bandwidth without considering the base stations. In the 5G edge based vehicular networks, the communication between the vehicles and edge devices can be done through D2D communication or mmWave technology.

### 3.3. Adversary Model

In this section, we describe the main attacks which an malicious user might conduct in the absence of this protocol EDM reporting scheme.

- A malicious vehicle can try to send EDM files when he is not enrolled for participation.
- A malicious user or vehicle can try to know the identity of the vehicles that reported the EDM file.
- A malicious vehicle can try to get the raw content of EDM which were sent through the network.
- A malicious vehicle can try to attack one or several edge nodes and try to process the EMD by impersonating a given edge node.
- A number of attackers (within or without the participating group) can try to jeopardize the whole network through a denial of service attack.

### 3.4. Security Objectives

We outline the security goals which the proposed scheme needs to achieve:

- Identity privacy preservation: the identities of the vehicles that report the EDMs should be preserved.
- Authentication: each vehicle that is involved in sending the EDMs should be authenticated before it is allowed to join the system.
- Confidentiality and integrity: the EDMs files generated and sent through the network should not be intercepted and modified during the communication.
- Key escrow resilience: the keys of the entities (vehicles) participating in EDM reporting should not be generated by a single entity. Thus, even if the RTA is comprised, the attackers can not disclose the signing keys of the vehicles.
- Access control: only the entities with matching policies should be able to retrieve the contents of EDMs.
- Non-repudiation and traceability: a vehicle should not deny any participation in the EDM reporting. In addition, RTA should be able to disclose the true identity of any entity if needed.
- Auditability: the EDMs records that are saved in the system should be securely kept and easily verifiable. Even if one node in the chain is compromised, the malicious user should not be able to modify and upload any EDM content.

### 3.5. Preliminaries

In this section, we describe the two main cryptographic techniques used to build our protocol. We first outline a lightweight signcrpytion technique which is not built on pairing operations, we also describe the underlying concepts for constructing a private blockchain.

#### 3.5.1. Signcryption Scheme without Bilinear Pairings

This scheme is made by six sub protocol namely Setup, SecretValue, Partialkeypair, keypair, Signcryption and DeSigncryption [30].

- $Setup(1^{\lambda })$: using a parameter λ, RTA runs the system to generate a master secret key mk and the parameters params.
- SecretValue(ID,params): a user runs the algorithm to return a secret value $V_{ID}$ using his/her identity ID.
- $Partialkeypair(params,V_{ID},ID)$: RTA runs the algorithm and returns the partial private key $y_{ID}$ and partial public key $D_{ID}$ using the user identity ID and the secret value $V_{ID}$
- $Keypair(D_{ID},y_{ID},params)$: the user generates the key pairs $\left(PK_{i},SK_{i}\right)$ using the partial key pairs $\left(D_{ID},y_{ID}\right)$.
- $Signcrypt(L,m,SK_{i},params)$: the user target a group of authorized receivers’ public keys $L=\{PK_{1},PK_{2},PK_{3},..,PK_{n}\}$ where *n* is a positive integer. Output a ciphertext δ on the message m.
- $Designcrypt(params,\delta ,SK_{i})$: using the system parameters params, the receiver’s private key $SK_{i}$ and the ciphertext δ, an authorized receiver recovers the message m.

#### 3.5.2. Private Blockchain

The private blockchain concept used in the paper is made by the following sub-phases, namely setup, initial stage, leader selection and block generation [21,22]:

- Setup: in this phase, different slot $\left\{ts_{1},ts_{2},ts_{3},\cdots \right\}$ are generated and a private ledger is attached with a one block for every time slot $ts_{i}$. In addition, a leader selection algorithm F(.) is assigned to each edge node.
- Initial stage: this is a first stake distribution phase when the first block also called genesis block is generated. The genesis block includes the edge nodes identities, public keys and stakes. The first block is assumed to have an empty blockheader and signcryption is generated on it.
- Leader selection: taking each time slot $ts_{i}$, the edge nodes identities, their public key, the probability of an edge node corresponding to its stake, this function output the node leader.
- Blockgeneration: the chosen leader generates a new block which is made by a block header, its stake, the number of EDM recorded. Note that the blockheader is made by a blockheader number, hash of previous blockheader, a merkle hash root along with a time stamp. For interested readers, the overall details can be found in [21,22].

## 4. Protocol Description

Our proposed protocol is made by five main sub-protocols: setup, participation agreement, EDM reporting, EDM collection and private blockchain generation

### 4.1. Protocol Setup

Our protocol assume that a regional traffic authority (RTA) manages the reporting of EDM messages, therefore both the vehicles and the edge nodes in the region are registered to the RTA. RTA first runs $Setup(1^{\lambda })$ to generates the parameters parameters $\left(G_{1},q,P\right)$ with the $G_{1}$ being a cyclic additive group of order *q* and a generator P over an elleptic curve that is defined on finite field $F_{w}$ where *w* is an integer chosen by RTA. RTA then selects a random $s\in Z_{q}^{*}$ as a master secret and generates the public key of RTA as $P_{RTA}=sP$. RTA selects five hash functions $H_{1}:{\left\{0,1\right\}}^{*}\to G_{1}$, $H_{2}:{\left\{0,1\right\}}^{*}\to G_{1}$, $H_{3}:{\left\{0,1\right\}}^{*}\to G_{1}$, $H_{4}:{\left\{0,1\right\}}^{*}\to G_{1}$, $H_{5}:{\left\{0,1\right\}}^{*}\to G_{1}$ and publishes the public parameters $\left(G_{1},H_{1},H_{2},H_{3},H_{4},H_{5},P_{RTA},P,q\right)$ to all the entities.

### 4.2. Participation Agreement

In order to participate in EDM reporting, the vehicles and the edge nodes are registered by the RTA. The registration of these entities is done as follows

- Step 1. Assume there is a vehicle inspection within a given period (12 or 18 months), the vehicle owner or user can express its desire to be part of EDM reporters. In this case, the vehicles does the following:
  1. A vehicle $v_{i}$ with its identity $5GID_{v_{i}}$ selects a secret $t_{i}\in Z_{q}^{*}$ and computes $V_{v_{i}}=t_{1}P$ and send $\left(5GID_{v_{i}},V_{v_{i}}\right)$ to RTA.
  2. Upon receiving $\left(5GID_{v_{i}},V_{v_{i}}\right)$, RTA choose a pseudonym for $5GID_{v_{i}}$ as $P5GID_{v_{i}}$, and keep the mapping table securely. RTA selects $d_{i}\in Z_{q}^{*}$ and computes $y_{i}=H_{1}\left(P5GID_{v_{i}},V_{v_{i}},d_{i}\right)+s\left(mod\right)w$ and $D_{i}=H_{1}\left(P5GID_{v_{i}},V_{v_{i}},d_{i}\right)P.$ Then RTA returns $\left(D_{i},y_{i}\right)$ to $v_{i}$
  3. $v_{i}$ receives $\left(D_{i},y_{i}\right)$ and checks if the equation $y_{i}P=D_{i}+P_{RTA}$ is correct. If yes, $v_{i}$ generates its public key $PK_{v_{i}}=D_{i}+H_{2}\left(P5GID_{v_{i}},V_{v_{i}}\right)V_{v_{i}}$.
  4. $v_{i}$ generates its private key $SK_{v_{i}}=H_{2}\left(P5GID_{v_{i}},PK_{v_{i}}\right)\left(y_{i}+H_{2}\left(5GID_{v_{i}},V_{v_{i}}\right)t_{i}\right)\left(mod\right)w$. The key pair of the vehicle $v_{i}$ is $\left(PK_{v_{i}},SK_{v_{i}}\right)$.
- Step 2. In the same way, RTA registers the edge nodes as follows:
  1. A edge node $Ed_{i}$ with its identity $ID_{Ed_{i}}$ selects a secret $m_{i}\in Z_{q}^{*}$ and computes $V_{Ed_{i}}=m_{1}P$ and send $\left(ID_{Ed_{i}},V_{Ed_{i}}\right)$ to RTA.
  2. After receiving $\left(5GID_{Ed_{i}},V_{Ed_{i}}\right)$, RTA selects $f_{i}\in Z_{q}^{*}$ and computes $a_{i}=H_{1}\left(ID_{Ed_{i}},V_{Ed_{i}},f_{i}\right)+s\left(mod\right)w$ and $F_{i}=H_{1}\left(ID_{Ed_{i}},V_{Ed_{i}},f_{i}\right)P.$ Then RTA returns $\left(F_{i},a_{i}\right)$ to $v_{i}$
  3. $Ed_{i}$ receives $\left(F_{i},a_{i}\right)$ and check if the equation $a_{i}P=F_{i}+P_{RTA}$ is correct. If yes, $Ed_{i}$ generates its public key $PK_{Ed_{i}}=F_{i}+H_{2}\left(ID_{Ed_{i}},V_{Ed_{i}}\right)V_{Ed_{i}}$.
  4. $Ed_{i}$ generates its private key $SK_{Ed_{i}}=H_{2}\left(ID_{Ed_{i}},PK_{Ed_{i}}\right)\left(a_{i}+H_{2}\left(ID_{Ed_{i}},V_{Ed_{i}}\right)m_{i}\right)\left(mod\right)w$. The key pair of the edge node $Ed_{i}$ is $\left(PK_{Ed_{i}},SK_{Ed_{i}}\right)$.

### 4.3. Emergency Driven Message Reporting

Whenever an emergency event such as land sliding occurs, $v_{i}$ performs the following:

- Composes EMD file as M={Dt,Ts,loc,file} representing the date, the time, the location and main file which has been captured. file could be a multimedia item such as pictures or audio files.
- $v_{i}$ generates a list of edge nodes that can recover the message, and in this case we adopt proximity protocol based on the location as described in [31]. $v_{i}$ generates $L=\{ID_{Ed_{1}},ID_{Ed_{2}},ID_{Ed_{3}},\cdots ,ID_{Ed_{n}}\}$ and make the signcryption on the event message as follows
  1. Computes $Q_{i}=PK_{Ed_{i}}+P_{RTA}$ with i=1,2,3,⋯,n
  2. Selects a integer $x\in Z_{q}^{*}$ and computes X=xP and $C_{i}=xH_{2}\left(PID_{Ed_{i}},PK_{Ed_{i}}\right)Q_{i}$ and $\alpha_{i}=H_{3}\left(C_{i},X\right)$ where i=1,2,..n
  3. Selects an integer $\xi \in Z_{q}^{*}$ and computes the polynomial $f\left(v\right)=\prod_{i=1}^{n}\left(v-\alpha_{i}\right)+\xi \left(mod\right)w$, which equals to $a_{0}+a_{i}v+..+a_{n-1}v^{n-1}$ for $a_{1}\in Z_{q}^{*}$
  4. Computes $k=H_{4}\left(\xi \right)$, $J=Enc_{k}(m||P5GID_{v_{i}})$ and $h=H_{5}(m||P5GID_{v_{i}},\xi ,a_{0},a_{1},..,a_{n-1},X)$
  5. Generates $h^{-1}$ that satisfy $hh^{-1}\equiv 1\left(mod\right)w$ and computes $z=h^{-1}\left(SK_{v_{i}}+x\right)\left(mod\right)w$
  6. Generates the cipher text $CT=<J,X,z,h,a_{0},a_{1},\cdots ,a_{n-1}>$ and send it to edge nodes.

### 4.4. Emergency-Driven Message Collection

Upon receiving the cipher text CT, an edge node $Ed_{i}$ does the following to recover the emergency warning

- Compute $C_{i}=SK_{Ed_{i}}X$ and $\alpha =H_{3}\left(C_{i},X\right)$
- Then computes $f\left(v\right)=a_{0}+a_{i}v+..+a_{n-1}v^{n-1}+v^{n}$ and $\xi =f(\alpha_{i})$
- Computes $k=H_{4}\left(\xi \right)$ and retrieve the message trough the decryption $Dec_{k}\left(J\right)=m\left|\right|P5GID_{v_{i}}$
- Also compute $h^{{}^{\prime }}=H_{5}\left(P5GID_{v_{i}},\xi ,a_{0},\cdots ,a_{n-1},X\right)$ and verifies if the equation $h=h^{{}^{\prime }}$ is correct. Otherwise, the emergency message is rejected
- Upon receiving the vehicle public $PK_{v_{i}}$,$Ed_{i}$ checks if the equation $hzP=H_{2}\left(P5GID_{v_{i}},PK_{v_{i}}\right)\left(PK_{v_{i}}+P_{RTA}\right)$. The correctness is as follows:
  $$\begin{array}{cc} \; & =hh^{-1}\left(SK_{v_{i}}+x\right)P\\ \; & =SK_{v_{i}}P+W\\ \; & =H_{2}\left(P5GID_{v_{i}},PK_{v_{i}}\right)\left(y_{i}+H_{2}\left(P5GID_{v_{i}},V_{v_{i}}\right)t_{i}\right)P+X\\ \; & =H_{2}\left(P5GID_{v_{i}},PK_{v_{i}}\right)\left(D_{i}+H_{2}\left(P5GID_{v_{i}},V_{v_{i}}\right)V_{v_{i}}+P_{RTA}\right)+X\\ \; & =H_{2}\left(P5GID_{v_{i}},PK_{v_{i}}\right)\left(PK_{v_{i}}+P_{RTA}\right). \end{array}$$

If yes, $Ed_{i}$ keeps the EDM message.

### 4.5. Private Blockchain Generation

We do assume that every edge node $Ed_{i}$ is a stakeholder having its proper stake that could be the number of valid EDM that $Ed_{i}$ has received. A private blockchain Chain is constructed as follows:

1. Assume that the time is divided into time slot $\left\{ts_{1},ts_{2},\cdots ,ts_{1}\right\}$ in which a block is attached to the ledger for each time sequence.
2. The initial block also called genesis block is generated as the first state distribution and it contains the edge nodes identities, their public keys, their stakes as $B_{gen}$ =< {ID${}_{Ed}$${}_{i=1}$ ${}^{R}$ }, ${\left\{PK_{Ed}\right\}}_{i=1}^{R}$ and ${\left\{ST_{Ed}\right\}}_{i=1}^{R}>$. We assume that first blockheader $B_{gen}$ to be empty.
3. Therefore, in a given area, each edge node $Ed_{i}$ set $C=B_{0}$ where $B_{0}$ is the genesis block
4. An edge node $Ed_{i}$ collects *n* EDM and verifies each EDM as shown in Section 4.4 by running $Designcrypt(params,\delta ,SK_{i})$. To choose a leader edge node $L_{Ed_{i}}$, the probability $p_{i}$ for being chosen should be relative to its stake that are in previous block.
5. $Ed_{i}$ runs a leader selection protocol F(.) [32] that input $<\{ID_{Ed}$${}_{i=1}$${}^{R}$}, ${\left\{PK_{Ed}\right\}}_{i=1}^{R},p_{Ed_{i}},st_{i}>$ representing respectively the edge nodes identities, their public key, the probability of the leader and the corresponding time slot with $p_{Ed_{i}}=st_{i}/\sum_{j=1}^{Ed_{i}}st_{Ed_{i}}$.
6. F(.) outputs a leader edge node $L_{Ed_{i}}\in \left\{Ed_{i},Ed_{2},Ed_{3},\cdots ,Ed_{n}\right\}$
7. To generate a block, the selected edge node $L_{E}d_{i}$ output a block $B_{ts_{i}}$ that corresponds to the time slot $ts_{i}$ with $B_{ts_{i}}=\left\{Num_{ts_{i}},H_{ts_{i}},MHR_{ts_{i}},t_{ts_{i}}\right\}$ representing respectively the number of the block, a hash corresponding to previous blockheader, merkle hash root corresponding to a merkle tree built using *n* EDM.
8. $Ed_{i}$ performs the update of its stake $st_{ts_{i}}$ and generates a signcryption on the entire message.
9. Finally add the block to the chain and send a notification to the entire network

## 5. Performance

This section is made by the security analysis of the proposed protocol, the experiment on private blockchain, the computational and communication cost along with the simulation.

### 5.1. Security Analysis

We provide in this section the analysis in regards to the security goals for the proposed scheme.

#### 5.1.1. Privacy Preservation

The communication within the proposed protocol is entirely based on anonymous interactions. While the vehicles engaged in reporting EDM are sending their messages, they make use of pseudonyms. RTA is the one and only entity that can map the real identity of a vehicle participating in the EDM reporting to its pseudonyms. As described in Section 4, when $v_{i}$ requests partial key pair by running the function $Partialkeypair(params,V_{ID},ID)$, it sends its real identity to RTA which generates a pseudonymous $P5GID_{v_{i}}$. Therefore it is infeasible for any entity inside or outside the network to know the real identity of the EDM participant except the RTA. This would require the adversary to access the database that maps the vehicles real identities and their pseudonyms.

#### 5.1.2. Authentication

In the proposed protocol, bad actors or malicious vehicles or any entity can not successfully engage in forging an EDM report because the authentication between a vehicle $v_{i}$ and an edge node $Ed_{i}$ is achieved through the signcryption function $Signcrypt(L,m,SK_{i},params)$ that is made on each message. Once an EDM is generated, $v_{i}$ makes signcryption of the message M={Dt,Ts,loc,file} by running $Signcrypt(L,m,SK_{i},params)$. Any entity needs to possess a valid private key $SK_{i}$ to be able to verify the correctness of the equation $hzP=H_{2}\left(P5GID_{v_{i}},PK_{v_{i}}\right)\left(PK_{v_{i}}+P_{RTA}\right)$. It is hard for an adversary to have the full private key of an edge node because it is partial generated both by the RTA and the edge node through the functions $Partialkeypair(params,V_{ID},ID)$ and $Keypair(D_{ID},y_{ID},params)$. The signcryption technique that was used to build the proposed protocol achieves unforgeability through the strong existential unforgeability against chosen plain text, selvi2008efficient. Therefore, we confirm that the proposed protocol achieves authentication property.

#### 5.1.3. Confidentiality and Integrity

The proposed protocol achieves the confidentiality and integrity of the EDM messages that are sent by the vehicles to edge nodes through the two in one technique that both provide encryption and digital signature in a single step. As shown in Section 4, the cipher $CT=<J,X,z,h,a_{0},a_{1},\cdots ,a_{n-1}>$ accomplishes the duties of message encryption and signature. A malicious user can not tamper with the integrity of the EDM because the signcryption phase transform the data into hash values $C_{i}=xH_{2}\left(PID_{Ed_{i}},PK_{Ed_{i}}\right)Q_{i}$ and $\alpha_{i}=H_{3}\left(C_{i},X\right)$ as described in Section 4. Thus, we guarantee that the proposed scheme achieves data integrity and confidentiality because the underlying technique is fully proved to satisfy security under adaptively chosen ciphertext [33].

#### 5.1.4. Key Escrow Resilience

The 4th industrial revolution projects a massive connectivity of devices to offer diversified services such as EDM reporting using the inbuilt vehicle sensors. In additional, 5G cellular networks were adopted in this work to provide effective latency. Security wise, key escrow resilience property needs to be achieved for applications within a massive connectivity environment. In the proposed scheme, the entities first generate a secret value by running SecretValue(ID,params) and RTA will then provide partialkey pair to the entities by computing $Partialkeypair(params,V_{ID},ID)$ function. The entities in our system compute their key pairs through the function $Keypair(D_{ID},y_{ID},params)$. Therefore, the proposed protocol achieves key escrow resilience.

#### 5.1.5. Access Control

In the current era with millions of devices connection, a single point failure should be avoided as much as possible. While the EDM are categorized to be safety related messages that contain sensitive data, multi receiver property (or access control) is a key point that need to be considered. In the proposed protocol, a vehicle $v_{i}$ selects a number of valid edge nodes, in this case, even in a scenario where a number of edge nodes have been compromised, the probability that the EDMs at least get to one receiver is higher. Therefore $v_{i}$ generates $L=\{ID_{Ed_{1}},ID_{Ed_{2}},ID_{Ed_{3}},\cdots ,ID_{Ed_{n}}\}$ and run $Signcrypt(L,m,SK_{i},params)$ to signcrypt the messages. Only valid receiver within the *L* can recover the EDM. Therefore, the proposed scheme achieves fine-grained access control by using attribute based encryption.

#### 5.1.6. Traceability and Non Repudiation

In the proposed system, when a valid user sends a fake EDM (probably for criminal profit), the edge node will discard the message because the signcryption correctness $hzP=H_{2}\left(P5GID_{v_{i}},PK_{v_{i}}\right)\left(PK_{v_{i}}+P_{RTA}\right)$ will not hold. However, $Ed_{i}$ will keep a log of pseudo identity of the vehicles. Thus, the vehicle can not deny its own pseudo identity. Additionally, in case of legal disputes, RTA consults its database that maps the identities and their pseudo identities to reveal the real identity of the vehicle. Therefore, we do confirm that the proposed protocol can achieve traceability and non repudiation of misbehaving entities.

#### 5.1.7. Auditability

The proposed scheme does achieve data auditability by building a private blockchain between the edges nodes. The achievement of this property can be summarized in three steps:

- The blockchain that is built in this scheme is private, any participant requires a permission or an invitation to join the private chain. In this case, it is infeasible for an malicious user to add bogus block to the chain.
- Each participant in the private chain keeps a replica of any appended ledger of emergency warning messages. This is crucial in case a crash occurs in any of the remote servers where the EDM are kept.
- Transactions immutability: It is hard for a malicious entity to tamper the EDM that is exchanged between the vehicles and the edges. In case of a legal dispute that require the thorough auditability of the EDM, the transaction immutability of blockchain can strengthen such services.

#### 5.1.8. Secure against Known Attacks

We describe in this section few well known attacks within the vehicular networks and how our proposed scheme can overcome them.

- Impersonation attack: as mentioned earlier, the malicious vehicles cannot succeed to impersonate a legitimate vehicle because the authentication between a vehicle $v_{i}$ and an edge node $Ed_{i}$ is achieved through the signcryption function $Signcrypt(L,m,SK_{i},params)$ that is made on each message. Once an EDM is generated, $v_{i}$ makes signcryption of the message M={Dt,Ts,loc,file} by running $Signcrypt(L,m,SK_{i},params)$. Every participating vehicle needs to possess a valid private key $SK_{i}$ to be able to verify the correctness of the equation $hzP=H_{2}\left(P5GID_{v_{i}},PK_{v_{i}}\right)\left(PK_{v_{i}}+P_{RTA}\right)$. Based on the hardness of the DL problem, the signature provided on the message cannot match the verification and the message will be discarded. Thus, it is almost impossible to perform an impersonation attack in our proposed scheme
- Masquerade attack: suppose a malicious user eavesdrops an EDM message and tries to know the EMD contents. That malicious user can not tamper with the integrity of the EDM because the signcryption phase transforms the data into hash values $C_{i}=xH_{2}\left(PID_{Ed_{i}},PK_{Ed_{i}}\right)Q_{i}$ and $\alpha_{i}=H_{3}\left(C_{i},X\right)$ as described in Section 4. Therefore, the malicious user cannot learn any useful information from the eavesdropped message nor reveal the identity of the message owner.
- DDoS attack: our scheme is able to resist against DDoS attacks either launched by legitimate or illegitimate vehicles. Assume an illegitimate vehicle tries to send multiple EDM to a given edge node, as demonstrated in the impersonation attack, those EDM will be discarded by the edge node because the message verification will not hold. In addition, assume a legitimate vehicle is generating excessive EDM to cause a DDoS attack, in that scenario, the edge nodes will use the time stamp on any EDM given message to predict the frequency of message compared to other users because every EDM message contains a time stamp as shown in message content as M={Dt,Ts,loc,file} representing respectively the date, the time, the location and file which could be a multimedia item such as pictures or audio files. Therefore, the messages from the suspicious user can be discarded.

### 5.2. Computational Cost

In this section we provide the analysis of the proposed protocol in terms of generating the EDM by the vehicle and the recovery of the message by the dedicated node. We performed the benchmark using a desktop of Core i7 3.5-GHz,16GB RAM with a crypto ++ library [34] with 6 as the embedded degree and G and *q* equivalent respectively to 161 bits and 160 bits. We mainly focused on the following main operations; point scalar multiplication, modular exponentiation and bilinear pairing. These operations dominate the process of sending and receiving the emergency messages. Table 1 shows the cost of the main cryptographic operations. A vehicle $v_{i}$ after generating the EDM, it performs $\left(T+1\right)T_{m}+nT_{p}$ to signcrypt a emergency message while the designcrypt operation requires $2T_{m}+T_{p}$ as shown in Table 2. As mentioned, there are several articles that addressed security solutions for BSM messages but few have addressed the EDM. BSM content being a predefined with limited content, the size of the BSM is supposed to be small and constant. As shown in this recent survey on secure protocol for vehicular communications [19], we compared the proposed protocol with the protocol in [20] as shown in Table 3.

**Figure 2:**
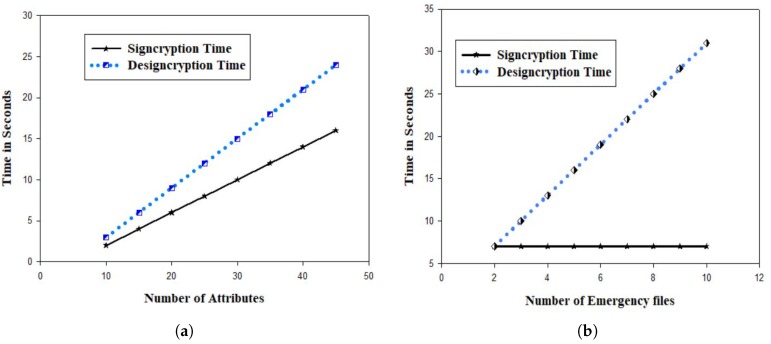
Signcryption/Designcryption time versus Number of Attributes (**a**) and Signcryption/Designcryption time versus Number Emergency Files (**b**).

We further considered two main elements than can effect the complexity of the whole scheme. First we investigated the number of attributes that can be associated with a given policy. Assume a user $v_{i}$ wants to share his emergency files with five governmental agencies. For instance, the emergency files contains few pictures of a land sliding scene. Those pictures can both be used by the ambulance team, the evacuation, the police and any other. Therefore the access policy might contain a number of attributes. In our simulation scenario we considered a range of attributes varying from 0 or 50, range=[0–50]. We then investigate the time needed for signcryption and designcryption based on the number of attributes in a given access policy. It is obvious that the obtained results are increasing gradually based on the number of attributes. For an average number of 30 attributes, the designcryption time was 17 s as shown in Figure 2a. Though the results are not very competitive, they are still feasible especially for edge nodes that have considerable computing capabilities. Also, we investigated the time needed for signcryption and designcryption when the number of files is fixed. The cost of encryption for one of two files was constant since we assume that the files (assume three separate images taken from different angles) are encrypted using a similar access policy. On the other hand, the decryption phase took much more time due to reconstruction of the secret value using Lagrange algorithm. As shown in Figure 2b, for a maximum folder of 10 files, we have a decryption cost of around 32 s. Since the decrypting devices could be servers or computing gadgets with sufficient communication power, the obtained decryption is acceptable for non real-time scenarios such as EDM reporting.

There are a considerable number of schemes within [19], however, these protocols are built based on expensive operations that compromise their efficient even if these protocols address BSM scenarios. For EDM, the size can be very important with the multimedia contents that can be added, thus a lightweight protocol could be more efficient. In additional, the protocols in the survey are not decentralized to offer immutability of transactions, however we achieve this property in our scheme by using private blockchain.

### 5.3. Communication Cost

In this section, we provide the communication cost of the proposed protocol. We first computed the overhead caused by the additional cryptographic primitives that were added on the raw message. We did not consider the element in the EDM since this can vary in real life application based on the multimedia content within an EDM. As mentioned in [35], in pairing operations, the size of elements equals to 64×2=128 bytes while for the ECC based operations, the size of the elements are equal to 20×2=40 bytes. As seen in the construction of the proposed, our scheme is not built based on pairing operations, and as described in Table 2, the size caused by security primitives are 80 bytes for the proposed protocol.

### 5.4. Private Blockchain Evaluation

We perform the experiments for the private blockchain that was built based on the edges nodes. Our experiment considers seven settings. As shown in Table 4, we considered a scenario that can generate 5 to 35 blocks. In the three first settings, we assumed 10 edge nodes while we considered 15 edges nodes in the four last settings. We computed the average time within our seven steps including the time required from system setup to the generation of the block. As seen in Table 4, the additional cost caused by the generation of block is not very heavy for a 5G cellular network, in the same time this technique offers immutable transactions with EDM auditability even if one or several edge nodes crash. We can see from the table that an edge node can create a new block to the added on the private blockchain with a cost of 0.056 s during the seventh step.

### 5.5. Simulation

In this section we provide the simulation that focus on the network performance of the proposed protocol. To achieve this, we made use of VANETSIM 2.02 that offers simulation for vehicle mobility and NS-3 was considered as a tool for network simulation. In our simulation, we considered a 5G functional network that can achieve a connection speed of 1.2Gb/s as reported and confirmed in several reports [36]. We focused on analyzing the performance of well known block ciphers techniques that vehicles can choose as symmetric encryption *k* as shown in Section 4. These algorithms were TWOFISH/CTR with 256 bit key and speed of 147 MB/s, then SERPENT/CTR that has 256 bit key with a 65 MB/s speed and lastly the famous AES/CBC of 256 bit key for a 455 MB/s as speed.

**Figure 3:**
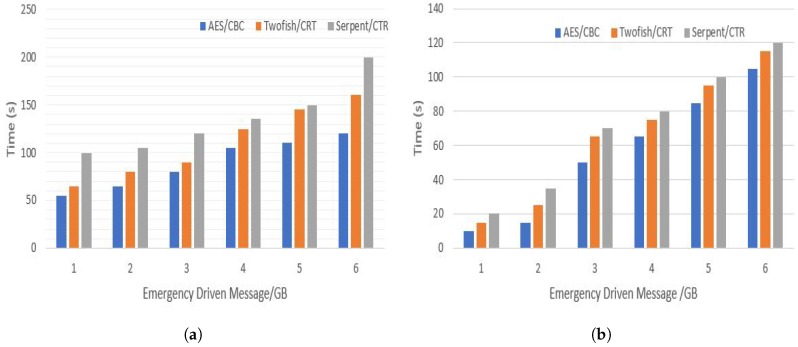
Overall time overhead (**a**) and signcryption time overhead (**b**).

The rest of the parameters that were considered in our simulation are described in Table 5. Our simulation mainly focus on the size of the EDM because till now we cannot tell what would be the real size of EDM, therefore using a 5G benchmarked connection, we investigated the performance of signcryption of EDM based on different sizes. The size of an EDM message varies between 1 to 6 Gigabytes. Figure 3b shows that the time needed by a vehicle to signcrypt an EDM, ranges between 20 to 40 s for an EDM that has a size of 2 GB. In addition, we investigated the overall time to signcrypt and designcrypt an EMD as shown in Figure 3a, we still found that as long as an EDM does not go beyond 2 GB of size, the overall time is not that much when we consider the 5G projected features. In this case, the highest record which corresponds to Serpent/CTR algorithm is around 100 s.

## 6. Conclusions

In this paper, we presented a secure and blockchain based EDM protocol for 5G-enabled vehicular edge computing. To provide scalability and latency for the proposed scheme, we adopted a 5G cellular architecture due to its projected features compared to 4G long-term evaluation (LTE) for vehicular communications. We considered an edge computing architecture to provide local processing of EDM in order to improve the response time. We made use of lightweight multi-receiver signcryption scheme without pairing that offers lightweight consuming operations, security, privacy and access control. To keep EDM records into a distributed system for reliability and auditability, we constructed a private blockchain using the edge nodes. The performance analysis of the proposed protocol in terms of security analysis, communication, computational and simulation confirms the efficiency of the protocol.

## Figures and Tables

**Table 1:** Measurement of cryptographic operations.

Notation	Operations	Time (ms)
$T_{b}$	Bilinear pairing	4.5
$T_{m}$	Point scalar multiplication	0.6
$T_{p}$	Point adddition on ECC	0.047
$T_{e}$	Exponentiation	3.9
$T_{as-dec}$	Asymmetric decryption	0.61
$T_{s-enc}$	Symmetric encryption	0.51
$T_{s-dec}$	Symmetric decryption	0.55
$T_{h}$	Execution time of a general hash function	0.0001

**Table 2:** Computational cost of signcrypt and designcript (*n* is number of receiver).

Phase	Operation
Signcryp an EDM	$\left(T+1\right)T_{m}+nT_{p}$
Designcryp an EDM	$2T_{m}+T_{p}$

**Table 3:** Comparison Performance of Proposed Work and literature.

Scheme	Lightweight	Traceability	Tamper Proof	Privacy	Decentralization	IoT Friendly
Liu et al., [20]	Low	YES	Low	Yes	NO	NO
Proposed Framework	High	YES	HIGH	YES	HIGH	HIGH

**Table 4:** Analysis of Edge node made private blockchain (/second).

Phase	No Trans/Block	No of ED	Initialization	Request	Response	Matching	Updating
1	5	10	0.022	0.44	0.15	1.89	0.0022
2	10	10	0.022	0.61	0.26	2.56	0.0089
3	15	10	0.022	0.98	0.63	2.29	0.014
4	20	15	0.045	1.44	0.89	3.51	0.031
5	25	15	0.045	1.79	1.25	4.01	0.056
6	30	15	0.045	2.14	1.67	4.98	0.17
7	35	15	0.045	4.12	3.90	6.67	0.56

**Table 5:** Setting of simulation parameters.

Tools/Parameter	Value/Specification
Mobility generation tool	VANETSIM 2.02
Network Simulation tool	ns-3
Data Rate	1.2 GBps
Number-of-vehicle	200
Number-of-edge nodes	40
Distance between two edge nodes	150 m
Simulation time	100 min
Wireless protocol	802.11a
Departure interval	180 s
RSU/Edge radius	800 m
mobility model	shortest path
Range of EDM size	(1–6 GB)
